# Effects of Calcareous Marine Algae on Feedlot Performance, Carcass Traits, Nutrient Digestion and Enteric Methane Emissions of Feedlot-Finished Nellore Heifers

**DOI:** 10.3390/ani16071024

**Published:** 2026-03-27

**Authors:** Igor Gomes Fávero, Ana Claudia Degli Exposti, Felipe Martins Fávero, Júlia Mara Campos de Souza, Antonio Pereira de Barros Neto, Robert Michael Boddey, Bruno Grossi Costa Homem, Lorenna Machado, Daniel Rume Casagrande, Erick Darlisson Batista

**Affiliations:** 1 Department of Animal Science, “Luiz de Queiroz” College of Agriculture (ESALQ), University of São Paulo, Piracicaba 13418-900, SP, Brazil; igor.favero@usp.br (I.G.F.); ana.exposti@usp.br (A.C.D.E.); antonioneto.zootecnista@gmail.com (A.P.d.B.N.); 2 Department of Animal Science, Federal University of Lavras (UFLA), Lavras 37200-900, MG, Brazil; felipe.favero@estudante.ufla.br (F.M.F.); jmaracamposs@gmail.com (J.M.C.d.S.); danielcasagrande@ufla.br (D.R.C.); 3 Department of Soil Science, Federal Rural University of Rio de Janeiro (UFRRJ), Seropédica 23897-000, RJ, Brazil; boboddey@gmail.com; 4 Department of Animal Sciences, Federal University of Viçosa (UFV), Viçosa 36570-900, MG, Brazil; bruno.c.homem@ufv.br; 5 College of Marine and Environmental Sciences, James Cook University, Townsville, QLD 4811, Australia; lorennamdf@hotmail.com

**Keywords:** finishing phase, natural additive, ruminants, sustainability

## Abstract

Global population growth has intensified the demand for productive and sustainable livestock systems. *Lithothamnium calcareum*, a calcareous marine alga, has been investigated as a natural feed additive for cattle diets. This study evaluated the effects of *L. calcareum* supplementation on performance, carcass traits, nutrient digestibility, nitrogen metabolism, urinary and fecal pH, and enteric methane emissions in Nellore heifers during the finishing phase. Thirty-six heifers (BW = 268.8 ± 7.3 kg) were assigned to individual pens in a completely randomized design and fed *ad libitum* diets (25:75 forage-to-concentrate ratio, DM basis). Treatments were: (1) sodium bicarbonate (110 g/heifer/day) and (2) *L. calcareum* (60 g/heifer/day). The 96-day trial included 12 days of adaptation and 84 days on the finishing diet. Methane emissions were measured using the sulfur hexafluoride (SF_6_) tracer technique. *L. calcareum* did not affect performance, carcass traits, nitrogen metabolism, or apparent total tract digestibility (all *p* ≥ 0.106), but reduced urine pH (*p* ≤ 0.001) and tended to lower methane emissions (−8.2%; *p* = 0.079). Thus, *L. calcareum* appears to be a viable natural alternative to sodium bicarbonate in finishing diets for Nellore heifers, maintaining productive performance and potentially reducing enteric methane output.

## Simple Summary

The growing demand for efficient beef production requires new strategies that keep animals productive under high-grain feeding conditions. One of the main concerns in modern cattle feeding is the frequent use of chemical additives and antibiotics, which has increased public interest in natural and environmentally friendly solutions. This study evaluated a calcareous marine algae called *Lithothamnium calcareum*, a natural source of calcium and other minerals, as a possible replacement for the traditional buffering compound sodium bicarbonate in beef cattle diets. The experiment was conducted with Nellore heifers housed in individual pens for 96 days and fed high-grain diets containing either sodium bicarbonate or the calcareous marine algae. The heifers receiving the algae showed similar feedlot performance compared with those receiving sodium bicarbonate. In addition, methane emissions tended to be 8.2% lower in the calcareous marine algae treatment (*p* = 0.079). Overall, this natural marine algae is a potential alternative additive that maintains performance and may contribute to more sustainable beef production.

## 1. Introduction

Meeting the growing global demand for ruminant-derived products is an increasing challenge as the world population expands, intensifying pressure on livestock production systems to improve both productivity and environmental sustainability [1]. Brazil, which accounts for a substantial share of global beef exports and approximately 12% of the world’s cattle herd [2], faces growing pressure from consumers and regulatory bodies to reduce the environmental footprint of beef production [3].

Methane (CH_4_) is the predominant greenhouse gas (GHG) emitted by ruminant production systems, produced mainly through enteric fermentation of feed substrates in the rumen [4]. The concern over CH_4_ emissions is significant due to its average atmospheric lifetime of approximately 12.4 years [5] and its high global warming potential (GWP_100_ = 27.2), making it a major target for mitigation efforts [6].

In addition to environmental concerns, methane formation also represents an energy loss of up to 12% of gross energy (GE) intake, reducing the efficiency with which cattle convert feed into productive outputs [7]. Therefore, strategies that reduce CH_4_ emissions may improve energy utilization and animal productivity. Studies, including those by Beauchemin et al. [8] and Knapp et al. [9], indicate that dietary strategies targeting CH_4_ reduction can improve ME availability, resulting in increased body weight gain (BWG) and improving feed efficiency. These findings highlight the potential of CH_4_ mitigation to enhance cattle productivity by reallocating this excess conserved energy toward growth.

Some nutritional strategies, such as the use of ionophores, have been widely used to improve feed efficiency and modulate rumen fermentation in high-concentrate diets [10]. Despite their effectiveness, the use of antibiotics as growth promoters may be limited in the future due to social and regulatory pressures [11] associated with the potential development of antibiotic-resistant pathogens [12]. For example, the European Union and Denmark banned the use of ionophores as growth promoters in animal production in 2006 and 1999, respectively [13,14]. Such restrictions have stimulated the search for natural feed additives capable of supporting ruminal stability without relying on antibiotics.

Among natural alternatives, several algae species, including the calcareous red alga *Lithothamnium calcareum*, have been explored due to their mineral composition and buffering properties that positively influence fermentation in vitro [15]. *L. calcareum* has a honeycomb structure that results in the slow release of minerals in an acidic environment [16]. Its physicochemical characteristics may be beneficial to rumen fermentation by enhancing buffering capacity, where the release of a bicarbonate ion can react with one proton, while the release of a carbonate ion can neutralize two protons [16], which has been suggested to confer greater theoretical acid-neutralizing potential than sodium bicarbonate.

Previous work evaluating *L. calcareum* with monensin reported reductions in CH_4_ production when compared to essential oils [17]. Although an in vitro screening study reported that *L. calcareum* may influence methanogenesis [15], no in vivo experiments have evaluated its effects when supplied alone, without other additives, under feedlot conditions.

Thus, we hypothesized that supplementation with *L. calcareum* would maintain productive performance and nutrient utilization while potentially mitigating methane emissions. The objective of this study was to evaluate the effect of *L. calcareum* on enteric CH_4_ emissions, nutrient digestibility, nitrogen metabolism, urinary and fecal pH, and growth performance of Nellore heifers during the finishing phase.

## 2. Materials and Methods

The experiment was conducted at the Research Feedlot of the Department of Animal Science (DZO), College of Animal Science and Veterinary Medicine (FZMV), Federal University of Lavras (UFLA), in Lavras, State of Minas Gerais, Brazil. All experimental protocols and activities in this trial were approved by the Ethics Committee on Animal Use of UFLA (protocol number 019/21).

### 2.1. Experimental Design, Animals, and Housing

Nellore heifers (*n* = 36; 15 months of age, 268.8 ± 7.3 kg initial body weight [BW]) were allocated to 36 individual pens in a completely randomized design (CRD) experiment. Heifers were randomly assigned to concreted semi-covered pens (18 pens per treatment; 20 m^2^), each equipped with an individual feed bunk and water trough. Upon arrival at the feedlot, before the step-up diets, the heifers were offered a receiving diet containing 80% corn silage, 17% ground corn, 1.6% soybean meal, 0.4% urea, and 1.0% mineral mixture (DM basis) to allow for adaptation to the new environment. Prior to the beginning of the experiment, heifers were processed using the following protocol: dewormed (Treo^®^ Ace; Zoetis, Campinas, SP, Brazil) and vaccinated against rabies (Rai-Vet; Laboratório Bio-Vet S/A, Vargem Grande Paulista, São Paulo, Brazil), respiratory diseases, and leptospirosis (CattleMaster^®^ 4+L5; Zoetis, Campinas, SP, Brazil), and received prophylaxis for botulism and other ruminant diseases (StarVac^®^; Labovet Produtos Veterinários Ltda., Feira de Santana, Bahia, Brazil). After a 21-day adaptation period to the facilities and completion of the health protocol described above, the heifers were gradually adapted to experimental diets over 12 days using three step-up diets (four days for each step), with an incremental increase of 5% in concentrate (from 60% to 65% to 70%, respectively), until reaching the finisher diet containing 75% concentrate and 25% corn silage inclusion (DM basis). The experimental period lasted 96 days, consisting of 12 days for diet adaptation and 84 days for the finishing phase, which was divided into three periods (days 13–40, 41–68, and 69–96).

### 2.2. Dietary Treatments

The experimental treatment diets involved the inclusion of two different feed additives (i.e., calcareous marine algae [*L. calcareum*] and sodium bicarbonate) in the otherwise identical finishing ration fed to all heifers. The inclusion of corn silage was 25.0% (DM basis), and the remainder of the diets consisted of 63.3% ground corn, 5.8% soybean meal from a single lot, 1.2% feed-grade urea, and 2.0% of a feedlot premix without additives (Table 1). A commercial algal product (Beecomb^®^, SeaQ Corporation, Sant Cugat del Vallès, Spain) was supplemented at 0.9% DM of the diet, targeting an intake of approximately 60 g *L. calcareum*/heifer/day. Sodium bicarbonate was included at 1.8% DM of the diet, corresponding to approximately 110 g/heifer/day, representing twice the inclusion rate of the algal product due to its lower buffering capacity, and serving as a control treatment. All the diets were formulated to provide sufficient energy, protein, minerals, and vitamins to meet BR-CORTE [18] requirements for Nellore heifers in the finishing phase.

### 2.3. Feeding Management and Measurements

To prepare the concentrate, all ingredients were individually weighed using a fixed scale. The concentrate for each treatment was prepared weekly as a batch in a stationary feed mixer (Incomagri^®^, Itapira, SP, Brazil; 1000 kg capacity). At each feeding, the concentrate and corn silage were weighed separately for each heifer. Total mixed rations (TMRs) were manually mixed and offered ad libitum twice daily at 0700 and 1600 h based on the previous day’s intake, targeting 5% refusals. Samples of all ingredients in each concentrate batch and corn silage were collected daily throughout the study, while one sample of the leftovers from each pen was collected twice a week. A weekly representative diet composite sample was obtained and partially dried in a forced-air oven (55 °C for 72 h). The weekly DM content of corn silage was used to adjust the ingredient proportions in the diets if DM deviated by more than 3% from the average. These samples were ground through a 1 mm screen using a Willye Super-type mill (STAR FT-80; Fortinox; Marconi Ltd.a, Piracicaba, SP, Brazil), and the laboratory DM content of the samples was determined by drying at 105 °C for 16 h. Feed offered was recorded daily, and refusals were collected and weighed weekly; DMI was calculated after correcting both offered feed and refusals for DM content.

### 2.4. Animal Production and Carcass Traits

Once the heifers were assigned to their respective treatment groups, they were reweighed individually on days 0 and 96 of the experimental period, after a 16 h feed curfew. Body weight was also measured on days 13, 41, and 69 of the experimental periods, and shrunk body weight (SBW) was estimated using a non-linear equation developed for Zebu cattle to account for gut fill (0.8915 × BW^1.0151^; [18]). The ADG was calculated as the difference between the initial, intermediate, and final shrunk BW, divided by the number of days of each period or overall. The gain-to-feed (G:F) ratio was calculated as ADG divided by DM intake for each period and over the entire experimental period. At the end of the experiment, the heifers were transported to a commercial abattoir (Frigorífico Iper Ltda., Divinópolis, MG, Brazil) for carcass measurements. Pre-slaughter handling was conducted following good animal welfare practices, and slaughtering procedures followed strict guidelines established and regulated by the Sanitary and Industrial Inspection Regulation for Animal Origin Products in Brazil [19]. Dressing percentage was calculated based on the final hot carcass weight (HCW) and BW after fasting.

### 2.5. Urine and Fecal Sampling

Fecal grab samples were collected from the rectum of individual heifers each day within the three separate sample collection periods throughout the experimental period (i.e., days 27 to 32, 55 to 60, and 83 to 88). To ensure sample representation and homogeneous sampling, samples were collected at 0600 h, 0900 h, 1200 h, 1500 h and 1800 h across five consecutive days of each collection period. A sub-sample (10 g) of each sample was homogenized with an equal weight of deionized water (1:1, water/feces), and fecal pH was measured immediately after collection using a portable pH meter by placing the probe directly into the homogenate, as described by Branstad et al. [20] and Abeyta et al. [21]. Another sub-sample of feces was stored to prepare a composite sample representative of the entire collection period, which was later used for additional laboratory analyses.

Urine spot samples were collected concurrently, and urine pH was immediately measured using a portable pH meter (TEC-7p MPp, MS Tecnopon Equipamentos Especiais Ltda., Piracicaba, SP, Brazil). Daily aliquots of urine (2 mL) were acidified with 8 mL of 0.036 N sulfuric acid and stored at −20 °C. After each sampling period (described above), the frozen subsamples were pooled to form a composite sample. This composite sample was used to determine the concentration of purine derivatives for each heifer, which are considered indirect indicators of microbial protein synthesis in the rumen [22,23].

### 2.6. Apparent Total Tract Digestibility

The apparent total tract nutrient digestibility was assessed using samples of feed offered and refused and feces collected during the three five-day sample collection described in Section 2.5. Feed offered and residues (Section 2.3), and fecal samples (Section 2.5) were collected on each day of the collection period and oven-dried (55 °C), ground to pass through a 2 mm screen, and a subsample of approximately 50% of the ground material was further ground to pass through a 1 mm screen for subsequent chemical analysis. After conducting the studies, apparent total-tract digestibility of nutrients was estimated using indigestible NDF (iNDF) as an internal marker, according to:

$$\mathrm{Apparent}\;\mathrm{total}\;\mathrm{tract}\;\mathrm{digestibility}\;\left(\%\right)\;=\;100\;\times \;\left[1\;-\;\left(\;\frac{{\mathrm{iNDF}}_{\mathrm{diet}}}{{\mathrm{iNDF}}_{\mathrm{feces}}}\;\right)\left(\;\frac{{\mathrm{Nutrient}}_{\mathrm{feces}}}{{\mathrm{Nutrient}}_{\mathrm{diet}}}\;\right)\right]\;$$

where ${iNDF}_{diet}$ and ${Nutrient}_{diet}$ are the DM-based concentrations in the composite diet sample and ${iNDF}_{feces}$ and ${Nutrient}_{feces}$ are the concentrations (DM basis) in the composite fecal sample. Indigestible NDF was adopted because it represents the indigestible residue of the fibrous fraction and is widely used as an internal marker to estimate fecal output and apparent total-tract digestibility when total fecal collection is not performed [24].

### 2.7. Enteric Methane Emission

Enteric CH_4_ emission was measured from individual heifers using the sulfur hexafluoride (SF_6_) tracer gas technique [25] in each of three separate collection periods which were immediately prior to the fecal and urine sample collection periods (Section 2.5). One month prior to the first collection period, a small brass permeation tube was orally administered into the rumen to allow the tracer gas to equilibrate. Prior to rumen insertion, SF_6_ permeation tubes were pre-conditioned and calibrated in a temperature-controlled oven at 39 °C for 6 weeks to determine individual SF_6_ release rates. The release rate for each tube (QSF_6_) was calculated from the change in tube weight over time and expressed as ng/min. Permeation tube release rates ranged from 961.8 to 1891.5 ng/min. Five days prior to the first gas collection period, the heifers were fitted with gas collection halters to acclimate to the collection apparatus prior to sample collection. The setup included a 0.127 mm stainless steel capillary tube halter, and a 15 μm in-line filter was placed on the heifer’s head and connected to an evacuated sampling vessel [26]. Before the experiment, sampling yokes made of polyvinyl chloride were attached to a vacuum pump in the laboratory to create negative pressure (approximately −13.15 psi). As the vacuum in the sampling vessel slowly dissipated, the negative pressure continuously drew in the air sample around the animals’ mouth and nose. The gas samples were obtained continuously through a capillary tube connected to a collecting container placed on the neck of each heifer. Yokes were removed from heifers at 0600 h each day with the sampled gas representative of gases present in breath samples collected over the previous 24 h period. Additional PVC yokes were placed near the experimental feedlot to monitor the ambient daily concentration (“basal concentration”) of CH_4_ and SF_6_ during each sampling period.

After collection of gas samples from yokes, pure nitrogen was added (approximately 1.5 psi above ambient) to the yokes, and CH_4_ and SF_6_ concentrations were analyzed using a single gas chromatograph (Shimadzu^®^ GC-2014, Kyoto, Japan). The chromatograph was equipped with a flame ionization detector (FID) and a megabore column (0.53 mm, 30 m) Plot HP-Al/M (for CH_4_), and a micro-electron capture detector (μ-ECD) and a megabore column HP-MolSiv (for SF_6_), with two 0.5 cm^3^ loops coupled to two six-way valves. The calibration curves were established using gas standards certified by White Martins (Praxair; Rio de Janeiro, RJ, Brazil), with concentrations in ppt (54 ± 9, 97 ± 9, and 954 ± 98 ppt) for SF_6_ and in ppm (0.996; 4.98; 10.36, and 51.57 ppm) for CH_4_, according to Johnson et al. [26]. The CH_4_ flux released by the animal was calculated from the SF_6_ flux, correlating the results to the known rate of tracer release in the rumen [26], following the equation:

$${\mathrm{CH}}_{4\;}\mathrm{emission}\;=\;{\mathrm{QSF}}_{6}\;\times \;\left\{\frac{[{\left({\mathrm{CH}}_{4}\right)}_{y}\;-\;{\left({\mathrm{CH}}_{4}\right)}_{b}]}{[{\left({\mathrm{SF}}_{6}\right)}_{y\;}-\;{\left({\mathrm{SF}}_{6}\right)}_{b}]}\right\}$$

where CH_4_ emission = CH_4_ emission rate per animal; QSF_6_ = known SF_6_ emission rate from the capsule in the rumen; (CH_4_)_y_ = CH_4_ concentrations in the collection device; (CH_4_)_b_ = basal concentration of CH_4_; (SF_6_)_y_ = SF_6_ concentration in the collection device; and (SF_6_)_b_ = basal SF_6_ concentration.

The CH_4_ emissions were expressed as CH_4_ production (g/day), CH_4_ yield (g/kg DM intake) and CH_4_ intensity (g/kg ADG).

### 2.8. Sample Analysis

Samples of feed offered, including individual ingredients, feed residues and feces were ground in a knife mill to pass through a 2 mm screen with approximately 50% then subsequently ground through a 1 mm screen. Samples of each material ground through 1 mm sieves (feed offered, feed residues and feces) were analyzed according to the standard analytical procedures of the Brazilian National Institute of Science and Technology in Animal Science (INCT-CA; [27]) for DM content (dried overnight at 105 °C; method INCT-CA no. G-003/1), ash content (complete combustion in a muffle furnace at 600 °C for 4 h; method INCT-CA no. M-001/1), with organic matter (OM) calculated by difference, and neutral detergent fiber (NDF) corrected for ash and protein content (NDFap; using heat-stable α-amylase, omitting sodium sulfite, and correcting for residual ash and protein; method INCT-CA no. F-002/1), and nitrogen (N) content (Leco FP-528; Leco Corp., St Joseph, MI, USA). The crude protein (CP) content was calculated by multiplying the nitrogen content by 6.25 [28]. From samples processed through a 2 mm sieve, an internal marker (indigestible NDF [iNDF]) was used to estimate total fecal output. The iNDF content was determined as the residual NDF remaining after 288 h of ruminal in situ incubation using F57 filter bags (Ankom Technology Corp., Macedon, NY, USA), according to Detmann et al. [27] and Neves et al. [29]. Starch analysis followed the recommended INCT-CA procedure (method No G-007/1). Measurements were taken using a UV/visible spectrophotometer set to 630 nm.

The concentration of creatinine, allantoin, uric acid, and urea in urine was determined by spectrophotometry. Creatinine and allantoin were analyzed using the colorimetric methods of Bartels et al. [30] and Chen & Gomes [23], respectively. Uric acid and urea were measured with enzymatic kits. Excretion of purine derivatives was calculated from the sum of the amounts of allantoin and uric acid excreted in the urine.

Microbial crude protein synthesis in the rumen (MCP, g/d) was estimated according to the equations proposed by Barbosa et al. [22]. Absorbed purines (AP, mmol/d) were calculated as:

$$\mathrm{AP}\;=\;\left[\mathrm{PD}\;-\;\left(0.30\;\times \;{\mathrm{BW}}^{0.75}\right)\right]\;/\;0.80$$

where PD represents the urinary excretion of purine derivatives (mmol/d) and BW is body weight (kg).

Microbial nitrogen synthesis (N_mic_, g N/d) was then estimated as:

$$N_{\mathrm{mic}}\;=\;\left[\;\frac{70\;\times \;\mathrm{AP}}{0.93\;\times \;1000\;\times \;0.137}\;\right]$$

assuming a true intestinal digestibility of microbial RNA of 0.93 and a purine-N to total microbial N ratio of 0.137.

Microbial crude protein synthesis was calculated as:

$$\mathrm{MCP}\;=\;N_{\mathrm{mic}}\;\times \;6.25$$

Microbial protein synthesis efficiency was expressed as grams of microbial crude protein per kilogram of digestible organic matter intake (g MCP/kg DOM) and relative to crude protein intake (g MCP/kg CP).

### 2.9. Statistical Analysis

All statistical analyses were conducted using the MIXED procedure of SAS 9.4 (SAS Inst. Inc., Cary, NC, USA) with animals as the experimental unit. Treatments and feedlot periods were treated as fixed effects, and animals were treated as random effects. The statistical model for data analysis was as follows:

$$Y_{\mathrm{ijm}\;}=\;\mu \;+\;T_{i\;}+\;P_{j}\;+\;A_{m}\;+\;\varepsilon_{\mathrm{ijm}}$$

where: $Y_{\mathrm{ijm}}$ = variable from treatment *i,* feedlot period *j*, and animal *m*; μ = average; $T_{i}$ = fixed effect of treatment *i*; $P_{j}$ = fixed effect of period *j*; $A_{m}$ = random effect of animal *m*; $\varepsilon_{\mathrm{ijm}}$ = error.

Statistical analyses were conducted to compare treatment effects using Fisher’s protected least significant difference (LSD) test. A similar model was applied to data on urine and fecal pH, CH_4_ production, CH_4_ yield and CH_4_ intensity, incorporating time as a repeated measure. For these repeated measures, either compound symmetry or heterogeneous compound symmetry was used as the covariance structure, with the optimal choice determined by the lowest Akaike information criterion (AIC). When a significant treatment × time interaction was observed, simple effects were analyzed using the SLICE option within the LSMEANS statement. In the absence of interaction, and when the main effects of diet and time were significant, comparisons among diets and time points were also conducted using the LSD test. Statistical significance was defined as *p* ≤ 0.05, and results with 0.05 < *p* < 0.10 were interpreted as tendencies.

## 3. Results

### 3.1. Growth Performance

No period × treatment interactions were detected for feedlot performance (*p* = 0.560).

By design, initial BW did not differ between treatments (*p* = 0.923; Table 2). Heifers fed diets containing calcareous marine algae had similar final BW (*p* = 0.943), ADG (*p* = 0.998), DM intake (*p* = 0.780), and G:F (*p* = 0.636) compared to those receiving sodium bicarbonate. Additionally, carcass traits measured at slaughter, including HCW (*p* = 0.934) and dressing percentage (*p* = 0.729), were not affected by treatment.

The DM intake (*p* < 0.001) of heifers was lowest in the first period (6.48 kg/heifer/day), followed by the second period (7.02 kg/heifer/day), and highest during the third period (7.33 kg/heifer/day). The ADG of heifers was significantly higher (*p* < 0.001) during the third period of the experiment (1.31 kg/heifer/day) compared with the first and second periods, which averaged 0.849 kg/day and did not differ from each other. Consequently, G:F was influenced by period (*p* < 0.001), with the third period (0.18) having a more efficient feed conversion compared to the first and second periods (0.13).

### 3.2. Apparent Total Tract Nutrient Digestibility

Total tract nutrient digestibility of DM (*p* = 0.433), OM (*p* = 0.211), starch (*p* = 0.106), NDF (*p* = 0.310) and CP (*p* = 0.184) were unaffected by the inclusion of calcareous marine algae in the ration (Table 3).

### 3.3. Urine and Fecal pH

There was a trend for treatment x period interaction for urine pH (Table 4; *p* = 0.052). Urine pH was significantly lower (*p* < 0.001) for heifers consuming the ration containing the calcareous marine algae in the first (7.88 vs. 7.26), second (8.18 vs. 7.83), and third (8.26 vs. 7.91) collection periods compared to those consuming the ration containing sodium bicarbonate. Urine pH was also significantly lower (*p* < 0.001) during the first collection period (7.57) compared to the second (8.00) and third (8.09) collection periods, which did not differ.

No period × treatment interaction was detected for fecal pH (*p* = 0.524). Fecal pH was unaffected by dietary treatment at each of the collection periods and when averaged across the entire experimental period (Table 4; *p* = 0.134). However, fecal pH was significantly lower in the second collection period (5.94) compared to samples collected during the first (6.14) and third (6.15) collection periods.

### 3.4. Nitrogen Utilization

Nitrogen (N) intake was similar between heifers fed sodium bicarbonate and those fed calcareous marine algae (147.1g/d vs. 145.0 g/d; *p* = 0.758; Table 5). Fecal N excretion (54.55 vs. 48.73 g/d; *p* = 0.106), urinary N excretion (65.71 vs. 70.04 g/d; *p* = 0.483) and total N excretion (119.20 vs. 121.47 g/d; *p* = 0.780) were not affected by treatments.

Urinary excretion of purine derivatives did not differ significantly between the treatments. Specifically, allantoin (93.05 vs. 83.23 mmol/d; *p* = 0.277), uric acid (10.60 vs. 9.36 mmol/d; *p* = 0.200), and total purine derivative excretion (103.6 vs. 92.6 mmol/d; *p* = 0.269) showed no significant differences. Similarly, estimated microbial crude protein synthesis did not differ between treatments (*p* = 0.262), with values of 346.0 and 298.3 g/d for sodium bicarbonate and calcareous marine algae, respectively.

The efficiency of microbial protein synthesis did not differ between treatments when expressed relative to digestible organic matter intake (47.93 vs. 41.33 g/kg DOM; *p* = 0.360) or per unit of crude protein intake (361.8 vs. 307.5 g/kg CP; *p* = 0.318).

### 3.5. Enteric Methane Emissions

No period × treatment interactions were observed for CH_4_ production (g/day; *p* = 0.860; Table 6), CH_4_ yield (g/kg DMI; *p* = 0.790), or intensity (g/kg ADG; *p* = 0.734).

Methane production tended to be 8.2% lower (*p* = 0.079) in heifers fed rations containing calcareous marine algae compared to heifers consuming rations containing sodium bicarbonate. No differences were observed between the periods (*p* = 0.929).

Calculated CH_4_ yield (*p* = 0.212) and CH_4_ intensity (*p* = 0.312) did not differ between heifers fed rations containing the calcareous marine algae or sodium bicarbonate. Additionally, calculated CH_4_ yield did not differ across the three sampling periods (*p* = 0.141). However, calculated CH_4_ intensity was significantly lower (*p* < 0.001) in the third sampling period compared to the first two sampling periods, which did not differ from each other.

## 4. Discussion

*L. calcareum*, a red calcareous marine algae harvested from northern Europe and South America, is valued for its high concentrations of bioavailable calcium and magnesium. Previous studies have demonstrated the buffering potential of calcareous marine algae based on *L. calcareum* in cattle diets [16,31,32]. However, because ruminal pH was not measured in the present study, any inference regarding the occurrence of subacute ruminal acidosis should be made cautiously. Ruminal pH stability is particularly relevant in finishing cattle, as depressed dry matter intake (DMI) becomes evident when ruminal pH falls below approximately 5.6 [33]. Although ruminal pH was not directly measured, the similar DMI between treatments suggests that *L. calcareum* did not negatively influence ruminal conditions relative to sodium bicarbonate and that both additives exerted comparable effects on feed acceptability. These findings align with previous work in beef cattle [31], who also detected no significant effect on DMI when sodium bicarbonate was replaced with *L. calcareum.* Similar results were also reported in other studies involving dairy cows under high-concentrate diets [16,34], who also reported no significant effects on DM intake when replacing sodium bicarbonate with *L. calcareum* in cattle diets. Similarly, [17] reported no significant differences in ADG, DMI, or G:F when comparing the supplementation of calcareous marine algae in combination with monensin to monensin alone in beef cattle. These findings are consistent with the present study, in which performance parameters such as ADG and G:F ratio were also not affected by the inclusion of calcareous marine algae. Given that DM intake is a major determinant of cattle productivity, the absence of any differences in DM intake in the current experiment likely explains the lack of significant differences in ADG and carcass traits [35]. The results of the current experiment demonstrate that there are no negative effects of including *L. calcareum* in the diets of finishing beef heifers. Given the growing demand for sustainable and antibiotic-free livestock production, *L. calcareum* emerges as an additive for feedlot operations seeking to balance productivity with environmental responsibility.

The inclusion of *L. calcareum* did not significantly affect the apparent total tract digestibility of DM, OM, starch, NDF, and CP compared with sodium bicarbonate, indicating that its inclusion did not impair nutrient utilization and maintained digestive efficiency similar to conventional buffers. Although no statistical differences were detected, NDF and starch digestibility values were numerically higher in heifers receiving *L. calcareum.* One potential explanation relates to the physicochemical characteristics of this algae, which possesses a porous mineral structure capable of releasing Ca and Mg gradually [16]. This slow-release profile may contribute to maintaining more stable ruminal conditions compared with the rapid but short-acting neutralization provided by sodium bicarbonate. Stable ruminal pH is crucial for fibrolytic bacteria, which exhibit substantial reductions in activity when pH drops below approximately 5.7 [36,37,38,39]. Although amylolytic bacteria are more tolerant, their activity also declines as pH approaches the mid–5 range [40,41,42]. Thus, the numerical patterns in digestibility observed here are consistent with maintained digestive function, although the mechanism remains uncertain. Similar findings were reported by Montañez-Valdez et al. [43], who observed that calcified algae did not compromise NDF digestibility in high-concentrate diets. Overall, the digestibility outcomes indicate that *L. calcareum* maintains digestive efficiency and resulted in digestive responses comparable to those observed with sodium bicarbonate.

The pH results suggest that urinary and hindgut pH responses were not parallel, although the underlying physiological mechanisms cannot be determined from the present measurements. This difference may be partially explained by the distinct solubility of the compounds: sodium bicarbonate dissolves readily in water (approximately 9.6 g/100 mL at 20 °C; [44]), while calcareous marine algae exhibit limited solubility. *L. calcareum* is composed of several crystalline forms of CaCO_3_ (calcite, aragonite, vaterite) with lower solubility than sodium bicarbonate [45], resulting in slower mineral dissolution and limited systemic absorption [16]. These properties may help explain the lower urinary pH observed with *L. calcareum*, suggesting differences in renal acid handling rather than a direct reduction in systemic pH. Urine pH is generally used as an indicator of metabolic acid or alkali load [46]. The observed reduction in urinary pH may reflect alterations in systemic acid–base balance. The kidneys play a central role in maintaining blood pH homeostasis by increasing H^+^ excretion and conserving HCO_3_^−^ when systemic acidity increases, which leads to a decrease in urine pH [47]. Conversely, under alkaline conditions, increased bicarbonate excretion and reduced hydrogen ion secretion result in an increase in urine pH. Therefore, differences in urinary pH may reflect treatment-related physiological responses [48]. In this context, the lower solubility of L. calcareum may have contributed to a different physiological response pattern compared with sodium bicarbonate, although buffering dynamics were not directly measured. Conversely, no treatment effect was observed on fecal pH; however, this does not allow direct inference regarding ruminal pH stabilization. The period effect on fecal pH likely reflects normal microbial adaptation to high-concentrate diets over time rather than treatment differences.

The similar nitrogen intake observed between heifers fed sodium bicarbonate and those receiving *L. calcareum* is consistent with the comparable DMI and crude protein digestibility results. Although fecal nitrogen losses were numerically lower in heifers supplemented with *L. calcareum*, this difference did not translate into measurable changes in total nitrogen excretion. The lack of treatment effects on the urinary and total excretion of nitrogenous compounds aligns with the absence of changes in purine derivative excretion, including allantoin, uric acid, and total purines, which are widely recognized as indirect indicators of microbial protein synthesis [23]. These results suggest that *L. calcareum* supported microbial activity at levels similar to sodium bicarbonate. Notably, the efficiency of microbial protein synthesis did not differ between treatments, regardless of whether it was expressed relative to digestible organic matter (DOM) intake or crude protein (CP) intake. This result indicates that *L. calcareum* supplementation did not alter the efficiency of conversion of fermentable substrates into microbial protein. Because urinary nitrogen is the principal contributor to volatile nitrogen losses [49], the lack of treatment effects on urinary N excretion suggests that *L. calcareum* does not modify the environmental nitrogen footprint of finishing heifers. Overall, the nitrogen partitioning responses, together with stable intake and digestibility, indicate that *L. calcareum* supported nitrogen utilization similarly to sodium bicarbonate.

Although heifers supplemented with *L. calcareum* exhibited an 8.2% reduction in daily CH_4_ production compared to those receiving sodium bicarbonate, this difference represented a statistical tendency. Previous findings by Guerreiro et al. [17] demonstrated that the combination of *L. calcareum* with monensin significantly reduced enteric CH_4_ emissions compared to a blend of essential oils. In a recent meta-analysis, Cooke et al. [50] reported that monensin, when used alone, reduced CH_4_ production by approximately 15% across various dietary contexts, confirming its efficacy as a CH_4_ mitigation strategy in ruminants. These results suggest that while monensin independently reduces CH_4_ emissions, its association with *L. calcareum* may enhance this effect synergistically. Although the present study evaluated the impact of algae alone, the directional response observed suggests a potential role for *L. calcareum* in mitigating CH_4_. One possible mechanism of action may be related to its adsorptive capacity, as the porous, honeycomb-like structure of calcified marine algae [16] could facilitate the physical entrapment or adsorption of CH_4_ molecules within its mineral matrix. This structural feature, coupled with its slow-release mineral profile, may help stabilize the ruminal environment and facilitate the removal of CH_4_ or its precursors (e.g., H_2_) from the rumen ecosystem. Biochar studies have shown that its high surface area and microporosity can function as a sink for gases such as CH_4_ and NH_3_, thereby modulating microbial fermentation dynamics [51,52]. While direct evidence for similar mechanisms in marine algae is limited, the comparable physicochemical characteristics of *L. calcareum* suggest that such adsorptive processes may play a role in reducing enteric CH_4_ emissions. In this study, CH_4_ yield (g/kg DM intake) and intensity (g/kg ADG) were not significantly affected by treatment. However, a decline in CH_4_ intensity was observed over time, particularly during the final feedlot phase, likely due to improved feed efficiency and higher ADG, which are known to reduce methane output per unit of animal productivity [53]. In summary, although statistical significance was not achieved, the tendency should be interpreted cautiously and warrants further investigation in longer-term studies with greater power. Future work including direct measurements of ruminal pH and fermentation profiles would help clarify potential mechanisms.

## 5. Conclusions

The results of this study suggest that *Lithothamnium calcareum* resulted in responses similar to those observed with sodium bicarbonate when included in finishing diets for Nellore heifers, as it maintained feedlot performance, nutrient digestibility, nitrogen metabolism, and carcass characteristics under high-concentrate feeding conditions. Furthermore, the additive tended to reduce enteric CH_4_ emissions, although further research is required to confirm its efficacy with respect to these variables. Overall, these findings support the use of *L. calcareum* as an alternative functional and sustainable additive in finishing diets for beef cattle.

## Figures and Tables

**Table 1:** Ingredients and nutritional composition of the experimental diets.

Item	Sodium Bicarbonate	Calcareous Marine Algae
*Ingredient, % of DM*		
Corn Silage	25.0	25.0
Fine-ground corn	63.3	63.3
Soybean meal	5.8	5.8
Feed-grade urea	1.2	1.2
Feedlot premix ^1^	2.0	2.0
Sodium bicarbonate	1.8	-
Limestone	0.9	-
* Lithothamnium calcareum*	-	0.9
Kaolin ^2^	-	1.8
*Chemical composition, % of DM*		
DM, % as-fed	70.67	70.74
Crude Protein	12.66	12.76
Starch	57.83	56.62
NDF	23.17	23.44
Ether extract	3.33	3.33

^1^ Feedlot premix (Nutriltex Industria Zootecnica do Brasil Ltda., Lavras, MG, Brazil) supplied, per kilogram of diet DM, a minimum of 45 g P, 120 g Na, 60 g Mg, 35 g S, and 75 g of microminerals. ^2^ Kaolin is an inert clay/mineral used as a filler/carrier in the diet to standardize inclusion rates, with no intended nutritional or buffering effects.

**Table 2:** Effects of sodium bicarbonate and calcareous marine algae on performance and carcass traits of Nellore heifers during the finishing phase.

Item	Sodium Bicarbonate	Calcareous Marine Algae	SEM ^1^	*p*-Value
*Feedlot production*				
Initial BW, kg	268	269	7.2	0.923
Final BW, kg	368	368	9.1	0.943
Average daily gain, kg	0.902	0.902	0.0370	0.998
DM intake, kg	7.26	7.16	0.342	0.780
G:F	0.126	0.129	0.0056	0.636
*Carcass traits*				
HCW, kg	203	202	5.2	0.934
Dressing, %	55.7	55.5	0.45	0.729

^1^ SEM = standard error of the mean; BW = body weight; HCW = hot carcass weight; DM = dry matter; G:F = gain:feed.

**Table 3:** Effects of sodium bicarbonate and calcareous marine algae on the apparent total tract digestibility of nutrients by Nellore heifers during the finishing phase.

Item	Sodium Bicarbonate	Calcareous Marine Algae	SEM	*p*-Value
*Apparent total tract digestibility, %*				
Dry matter	60.64	62.06	1.682	0.433
Organic matter	61.97	64.23	1.659	0.211
Starch	84.87	87.89	1.268	0.106
Neutral detergent fiber	44.10	46.91	2.708	0.310
Crude protein	63.15	64.77	1.249	0.184

**Table 4:** Effects of sodium bicarbonate and calcareous marine algae on the pH of urine and feces collected from Nellore heifers during the finishing phase.

Item	Treatment		Period	SEM	*p*-Value		
	Sodium Bicarbonate	Calcareous Marine Algae			Treatment	Period	Treatment × Period
Urine pH							
d 35	7.88	7.26	7.57 ^a^	0.068	<0.001	<0.001	0.052
d 63	8.18	7.83	8.00 ^b^				
d 91	8.26	7.91	8.09 ^b^				
Overall	8.11	7.66	7.89				
Fecal pH							
d 35	6.16	6.12	6.14 ^a^	0.062	0.134	<0.001	0.524
d 63	5.95	5.93	5.94 ^b^				
d 91	6.22	6.07	6.15 ^a^				
Overall	6.11	6.04	6.07				

^a,b^ Statistical differences between means are denoted by superscript letters.

**Table 5:** Effects of sodium bicarbonate and calcareous marine algae on nitrogen metabolism and estimated microbial protein supply by Nellore heifers during the finishing phase.

Item	Sodium Bicarbonate	Calcareous Marine Algae	SEM	*p*-Value
N intake, g/day	147.1	145.0	6.96	0.758
*N excretion*				
Fecal, g/day	54.55	48.73	3.396	0.106
Urinary, g/day	65.71	70.04	4.322	0.483
Total N excretion, g/day	119.20	121.47	6.638	0.780
*Purine derivatives excretion*				
Allantoin, mmol/day	93.05	83.23	9.748	0.277
Uric acid, mmol/day	10.60	9.36	1.030	0.200
Total purine derivatives, mmol/day	103.6	92.6	10.77	0.269
*Microbial protein*				
MCP, g/day	346.0	298.3	45.98	0.262
*Efficiency of microbial protein synthesis*				
g/kg DOM	47.93	41.33	7.970	0.360
g/kg CP	361.8	307.5	60.00	0.318

**Table 6:** Effects of sodium bicarbonate and calcareous marine algae on enteric methane emissions from Nellore heifers during the finishing phase.

Item	Treatment		Period	SEM	*p*-Value		
	Sodium Bicarbonate	Calcareous Marine Algae			Treatment	Period	Treatment × Period
*Methane emissions*							
Production, g/day							
d 27	144.9	134.1	139.5	8.32	0.079	0.929	0.860
d 55	150.3	132.5	141.4				
d 83	146.6	137.9	142.3				
Overall	147.1	134.8	141.1				
Yield (g/kg DM intake)							
d 27	22.2	21.6	21.9	1.33	0.212	0.141	0.790
d 55	21.8	19.3	20.5				
d 83	20.4	19.1	19.7				
Overall	21.4	20.0	20.7				
Intensity (g/kg ADG)							
d 27	169.6	168.8	169.2 ^a^	10.67	0.312	<0.001	0.734
d 55	174.5	166.0	170.3 ^a^				
d 83	120.8	102.4	111.6 ^b^				
Overall	155.0	145.7	150.4				

^a,b^ Statistical differences between means are denoted by superscript letters.

## Data Availability

The data supporting the findings of this study are owned by the funding company and are not publicly available due to data ownership agreements. Data may be made available from the corresponding author upon reasonable request and with permission of the funding company.
